# Persistence of SARS-CoV-2 Antibody Response across Diverse Clinical Settings in Oman: Insights from a Prospective ELISA-based Study

**DOI:** 10.1016/j.ijregi.2025.100736

**Published:** 2025-08-22

**Authors:** Asma Al Balushi, Zainab Abdul Hameed, Hasina Al Bahri, Zaina Al Maskari, Ahlam Al Balushi, Iman Al Balushi, Sachin Jose, Adil Al Wahaibi, Amina Al-Jardani, Samira Al-Mahruqi, Mohammed Hamed Nasr, Emily Adams, Tom E. Fletcher, Iman Nasr

**Affiliations:** 1 Infectious Diseases Unit, Department of Medicine, Sultan Qaboos University Hospital, University Medical City, Muscat, Oman; 2 Internal Medicine Department, Royal Hospital, Muscat, Oman; 3 Internal Medicine Department, Al Nahdha Hospital, Muscat, Oman; 4 Department of Infection Prevention and Control and Occupational Safety, Royal Hospital, Muscat, Oman; 5 Department of Surgery, Khoula Hospital, Muscat, Oman; 6 Research Section, Oman Medical Specialty Board, Muscat, Oman; 7 Section of Epidemiological Surveillance, Center for Disease Prevention and Control, Muscat, Oman; 8 Central Public Health Laboratories, Center for Disease Control and Prevention, Muscat, Oman; 9 Oman Country Office, Global Health, The Association of Public Health Laboratories, Muscat, Oman; 10 Ministry of Health, Muscat, Oman; 11 Diagnostic Lead of the Centre for Drugs and Diagnostics, Liverpool School of Tropical Medicine, Liverpool, UK; 12 Department of Clinical Sciences, Liverpool School of Tropical Medicine, Liverpool, UK

**Keywords:** SARS-CoV-2, ELISA, COVID-19, Antibody response, Serology, Linear mixed effects model

## Abstract

**Objectives:**

The durability of SARS-CoV-2 antibody response varies with disease severity [1]. Comparative data across diverse clinical settings in the Gulf region are limited. This study assessed the persistence of SARS-CoV-2 immunoglobulin G (IgG) antibodies over 6 months among hospitalized patients, outpatients, and healthcare workers (HCWs).

**Methods:**

In this prospective cohort study, 356 confirmed COVID-19 cases (123 inpatients, 113 outpatients, and 120 HCWs) were enrolled. Serum IgG titers were measured using enzyme-linked immunosorbent assay (ELISA) at baseline (≤14 days post-symptom onset), 4-6 weeks, 3 months, and 6 months post-infection. Longitudinal antibody dynamics were analyzed using a linear mixed-effect model adjusting for patient group, age, comorbidities, and symptoms.

**Results:**

Inpatients were older, male, and had more comorbidities, including obesity 54.5% (67 of 123), diabetes mellitus 39% (48 of 123), and hypertension 39.8% (49 of 123), compared with outpatients and HCWs. Peak antibody titers were reached at 4-6 weeks, with gradual decline over the 6-month period after initial infection across all groups (*P* <0.001). Inpatients demonstrated significantly higher IgG titers at all time points (*P* <0.001).

**Conclusions:**

Severe COVID-19 infection, older age, and comorbidities were linked to stronger, more durable IgG responses. These findings provide essential baseline data on post–COVID-19 immunity in the Gulf region during early pandemic.

## Highlights

• Inpatients had the highest and most sustained SARS-CoV-2 immunoglobulin G (IgG) titers.
• Health care workers had higher titers than outpatients, likely due to repeated exposure.
• Advanced age and comorbidities were associated with higher antibody responses.
• Symptomatic patients mounted stronger antibody responses.
• Immunoglobulin G titers peaked at 4-6 weeks, declining gradually over 6 months post-infection.

## Introduction

COVID-19 infection, caused by SARS-CoV-2, was first identified in Wuhan, China in December 2019. In March 2020, the World Health Organization declared it a pandemic [1]. Affected individuals can be asymptomatic, have mild symptoms or a more severe form of acute respiratory distress syndrome (ARDS) and a hyper-inflammatory state with multi-organ involvement [2,3]. Reverse transcription polymerase chain reaction (RT-PCR) testing is widely used to confirm the diagnosis, with serological testing mainly used to assess immune response of affected individuals or in sero-prevalence studies [1,4].

Most infected individuals (80-90%) mount a measurable antibody response after infection, but the magnitude and durability vary widely based on disease severity and comorbid conditions [[5], [6], [7], [8]]. In a 2021 cohort study, Elslande *et al.* [9] found that 22.2% of asymptomatic individuals and 2.6% of severe cases did not seroconvert. Although seroconversion of immunoglobulin (Ig) A and IgM occurs earlier, within 2 weeks of symptoms onset, followed by a rapid decline over 2-4 weeks, IgG titers peak within weeks and decline gradually over a longer period [[5], [6], [7],^10^,^11^]. Comparative studies across diverse populations, including severe cases requiring admission, outpatients with mild symptoms and health care workers (HCWs) remain limited. The majority of existing studies have focused on specific populations, often with short-term follow-up, and there are no existing comparative data from the Middle East and the Gulf countries [8].

Our study aimed to evaluate the persistence and dynamics of SARS-CoV-2 IgG antibodies across diverse populations in Oman, including outpatients, inpatients, and HCWs, over a 6-month period post-infection. Using longitudinal modeling, we aimed to identify clinical and demographic factors associated with stronger and more durable antibody responses, providing essential baseline data for understanding post–COVID-19 immunity in the Middle East and the Gulf region during the early pandemic period.

## Methods and materials

### Study design and patients included

This was a prospective, observational cohort study conducted from July 2020 to May 2021.

A total of 356 COVID-19 cases, confirmed by SARS-CoV-2 real time PCR (RT-PCR) from nasopharyngeal swabs, were included. They were categorized into three groups: 123 inpatients, 113 outpatients, and 120 HCWs. Inclusion criteria were age ≥13 years and confirmed COVID-19 cases by SARS-CoV-2 RT-PCR, with no prior history of COVID-19 infection or vaccination at enrollment. Patients were recruited from two tertiary care hospitals in Muscat, Oman, the Royal Hospital and Al Nahdha Hospital, HCWs of both institutions, and surrounding population in the community. Outpatients were identified through a daily list of confirmed COVID-19 cases provided by the Center of Operations and Management for COVID-19 at the Ministry of Health, Oman. A dedicated team was assigned for home visits for data and sample collection.

Outpatients and HCWs had mild illness without oxygen requirement at diagnosis. Inpatients were hospitalized for moderate to severe COVID-19 illness requiring oxygen therapy or had other COVID-19–related organ involvement. Written informed consent was obtained for all participants.

### Sample collection and SARS-CoV-2 serology testing

Blood samples were collected at four time points: baseline (within 14 days of symptoms onset), 4-6 weeks, 3 months, and 6 months post-infection. Follow-up samples for enzyme-linked immunosorbent assay (ELISA) testing were obtained by arranging appointment visits at the mentioned hospitals. For HCWs, baseline samples were not available as they were enrolled after infection onset.

The SARS-CoV-2 IgG was assessed using Mologic IgG (Mologic, UK) ELISA at the Central Public Health Laboratories. The method of the test used was a semi-quantitative determination of IgG binding antibodies against the nucleoprotein (NP) and the spike 2 (S2) antigens. The test was performed according to the manufacturer’s instructions. Results are expressed by calculating a ratio using the following formula: sample optical density "OD" ÷ (cut-off control optical density "OD" 1.8) = sample ratio, and graded and interpreted as follows: ≥1.1 arbitrary units "AU" was considered positive, <0.8 AU was graded as negative, and ≥0.8 AU to <1.1 AU was interpreted as intermediate or equivocal. Equivocal results were repeated on the primary sample and, if still equivocal, then a second sample was collected and tested within 2 weeks. The assay was validated by St. George’s University of London, UK and Liverpool School of Tropical Medicine, UK in a large cohort of sequential samples and found to have excellent sensitivity and specificity of 91.1% and 98.6%, respectively [12,13].

### Statistical analysis

Continuous variables were presented as mean, median, SD, and interquartile range, whereas categorical variables were presented as frequencies and percentages. A comparison of continuous variables between two independent groups was conducted using the independent sample Student’s *t*-test, and comparison across three or more independent groups (e.g. inpatients, outpatients, and HCWs) were performed using one-way analysis of variance (ANOVA). Categorical variables were compared between groups using chi-square tests, where assumptions were met. For conditions with fewer than five total cases across all groups, statistical testing was not performed due to insufficient sample size and violation of chi-square assumptions. These conditions were reported descriptively.

ELISA titers were considered as the dependent variables and were converted into longitudinal format, with each patient having repeated measurements at four time points: baseline within 14 days of symptoms onset, 4-6 weeks, 3 months, and 6 months. A linear mixed-effect (LME) model was used to account for repeated measures within individuals by incorporating random intercepts per patient to control for within-subject correlation. Time was considered as a fixed effect, along with interaction terms between time and key covariates. Covariates included patients’ category (inpatients, outpatients, and HCWs); age group (13-40, 41-59, ≥60 years); gender; comorbidities, such as obesity, hypertension, diabetes mellitus, asthma, and chronic kidney disease; and symptoms, such as fever and cough.

The LME model was chosen due to its robustness in handling correlated repeated measures and its ability to accommodate randomly missing data. The models were fitted using the restricted maximum likelihood method (REML), and univariate and multivariate models were run; the results were reported as ratios of mean ELISA titers in each group. The 95% confidence intervals (CIs) were computed for each time point and subgroup. Statistical significance of interaction terms was examined to assess whether changes in antibody levels differed by subgroup characteristics, (*P* <0.05). All analysis was conducted using IBM SPSS Statistics (version 29.0), R software (version 4.1.2) and Jamovi (version 1.6.23.0). The mixed-effects regression models were performed using R (Ime4 package) [14]. ELISA titers were visualized using line plots with CIs for each subgroup.

## Results

### Demography

A total of 356 patients with confirmed COVID-19 were included in our study and divided into inpatients (123 of 356, 34.6%), outpatients (113 of 356, 31.7%), and HCWs (120 of 356, 33.7%). The majority were Omani 299 of 356 (84.0%: 95.9% of the inpatient group; 84.1% of the outpatient group; and 71.7% of the HCW group). Patients were stratified into three groups by age: 13-40 years, 41-59 years, and ≥60 years. Although 61.8% (76 of 123) of the inpatients were in the 41-59 years age group, the majority of the outpatients and HCWs were in the younger age group of 13-40 years (69% [78 of 113] and 69.2% [83 of 120], respectively; *P* <0.001). Inpatients were mainly males, 65% (80 of 123), whereas outpatient and HCWs were mainly females (54.9% [62 of 113] and 82.5% [99 of 120], respectively; *P* <0.001) (Table 1).

**Table 1:** Patients’ demographics and comorbidities by study groups.

Variable	Inpatients 123	Outpatients 113	Health care workers 120	
Demographics	n (%)	n (%)	n (%)	*P*-value
**Age**				
13-40 years	33 (26.8)	78 (69.0)	83 (69.2)	
41-59 years	76 (61.8)	29 (25.7)	35 (29.2)	**<0.001**^a^
≥60 years	14 (11.4)	6 (5.3)	2 (1.7)	
**Gender**				
Male	80 (65.0)	51 (45.1)	21 (17.5)	**<0.001**^a^
Female	43 (35.0)	62 (54.9)	99 (82.5)	
**Comorbidities**				
Obesity	67 (54.5)	11 (9.7)	9 (7.5)	**<0.001**^a^
Pregnancy	0 (0)	2 (1.8)	1 (0.8)	
Diabetes mellitus	48 (39.0)	14 (12.4)	7 (5.8)	**<0.001**^a^
Hypertension	49 (39.8)	6 (5.3)	13 (10.8)	**<0.001**^a^
Chronic kidney disease	8 (6.5)	2 (1.8)	1 (0.8)	
Chronic heart disease	7 (5.7)	2 (1.8)	2 (1.7)	
Chronic lung disease	12 (9.8)	2 (1.8)	4 (3.3)	
Chronic liver disease	0 (0)	1 (0.9)	0 (0)	
Chronic neurological disease	3 (2.4)	2 (1.8)	1 (0.8)	
Chronic hematological disease	1 (0.8)	1 (0.9)	0 (0)	
Cancer	1 (0.8)	2 (1.8)	2 (1.7)	
Organ or bone marrow recipient	2 (1.6)	1 (0.9)	0 (0)	

a Statistically significant; chi-square test used where expected counts ≥5. For variables with sparse data or fewer than five total cases across all groups, statistical testing was not performed, and results are presented descriptively. Abbreviation: n, number.

### Comorbidities

Comorbid conditions were more prevalent in inpatients than in outpatients and HCWs. Obesity was the most frequently observed condition: 54.5% (67 of 123) of inpatients compared with 9.7% (11 of 113) of outpatients and 7.5% (9 of 120) of HCWs (*P* <0.001). This was followed by diabetes mellitus (DM) (39.0% of inpatients [48 of 123], 12.4% of outpatients [14 of 113], and 5.8% of HCWs [7 of 120]) and hypertension (HTN) (39.8% of inpatients [49 of 123], 5.3% of outpatients [6 of 113], and 10.8% of HCWs [13 of 120]; *P* <0.001). Chronic lung diseases and chronic kidney disease (CKD) were also more frequent among inpatients. A full list of comorbid conditions across all groups is provided in Table 1.

### Symptoms

Fever was the most commonly reported symptom across all groups, occurring in 91.9% of inpatients (113 of 123), 50.4% of outpatients (57 of 113), and 56.7% of HCWs (68 of 120) (*P* <0.001). Inpatients more frequently experienced lower respiratory tract symptoms, including cough 84.6% (104 of 123) and shortness of breath 70.7% (87 of 123) than outpatients and HCWs (*P* <0.001). Conversely, upper respiratory tract symptoms, such as runny nose (38.1%, 43 of 113) and sore throat (35.4%, 40 of 113), were more common among outpatients (*P* <0.001). Headache was most frequently reported among HCWs (42%, 50 of 120; *P* <0.001). Detailed symptom frequencies for all groups are provided in Table 2.

**Table 2:** Patients’ symptoms by study groups.

	Inpatients 123	Outpatients 113	Health care worker 120	
Variable	n (%)	n (%)	n (%)	*P*-value
Fever	113 (91.9)	57 (50.4)	68 (56.7)	<0.001^a^
Sore throat	8 (6.5)	40 (35.4)	39 (32.5)	<0.001^a^
Runny nose	0 (0)	43 (38.1)	24 (20.0)	
Cough	104 (84.6)	42 (37.2)	44 (36.7)	<0.001^a^
Shortness of breath	87 (70.7)	9 (8.0)	9 (7.5)	<0.001^a^
Chills	3 (2.4)	4 (3.5)	3 (2.5)	
Nausea/Vomiting	38 (30.9)	16 (14.2)	3 (2.5)	
Headache	11 (8.9)	34 (30.1)	50 (42.0)	<0.001^a^
Rash	0 (0)	1 (0.9)	0 (0)	
Muscle/Joint pain	94 (76.4)	18 (15.9)	47 (39.2)	<0.001^a^
Loss of appetite	41 (33.3)	6 (5.3)	2 (1.7)	
Loss of smell	8 (6.5)	29 (25.7)	17 (14.2)	<0.001^a^
Fatigue	92 (74.8)	44 (38.9)	14 (11.7)	<0.001^a^
Seizure/ Other neurological symptoms	2 (1.6)	0 (0)	1 (0.8)	
Adult Respiratory Distress Syndrome/Pneumonia	25 (20.3)	0 (0)	2 (1.7)	

a Statistically significant; chi-square test used where expected counts ≥5. For variables with sparse data or fewer than five total cases across all groups, statistical testing was not performed, and results are presented descriptively. Abbreviation: n, number.

### ELISA results and antibody titers

**Figure 1:**
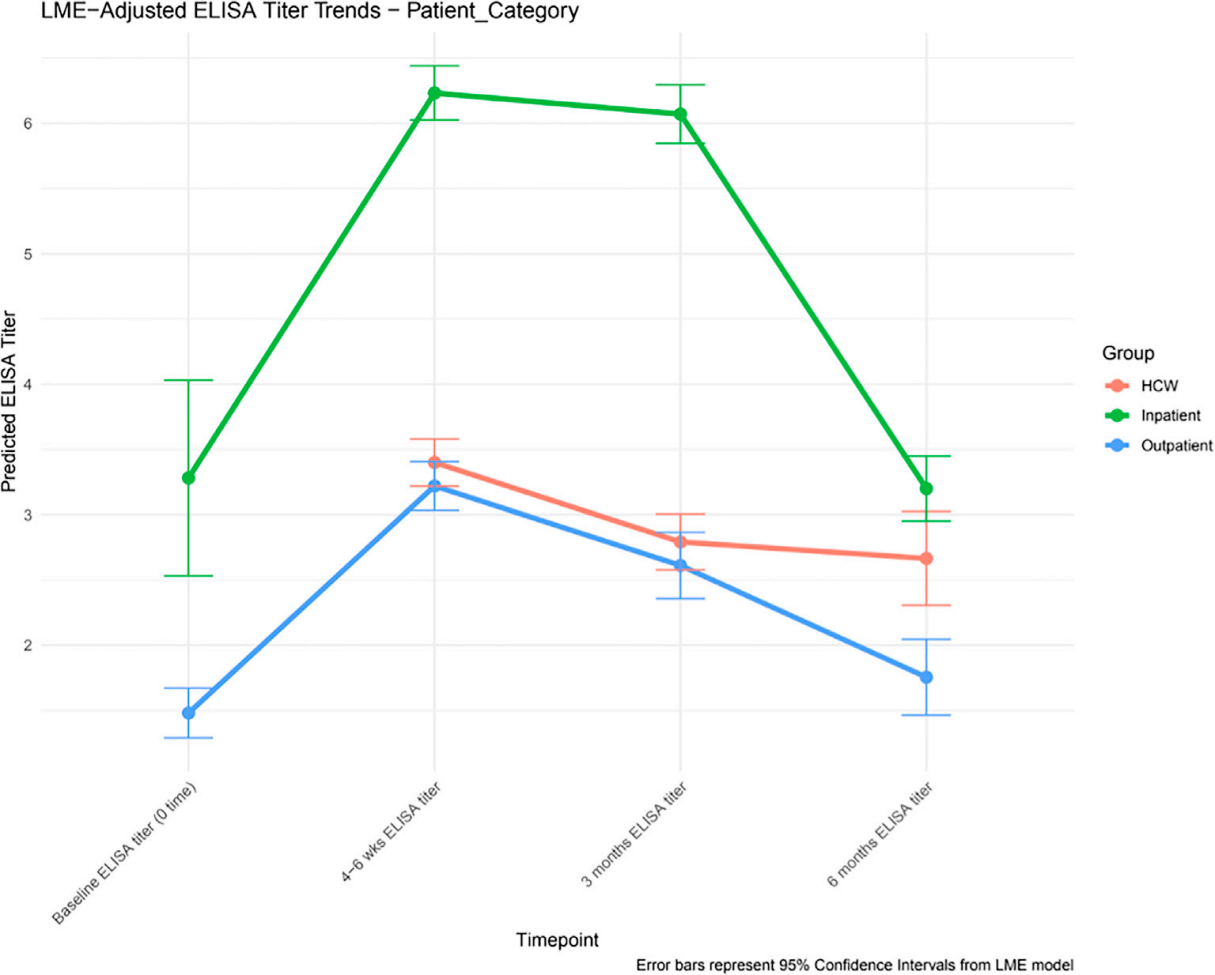
LME model: adjusted ELISA immunoglobulin G titer trends per patient category; inpatients, outpatients, and HCWs at four time points: baseline (within 14 days of symptoms onset), 4-6 weeks, 3 months, and 6 months post-infection. Antibody titers reached the peak at 4-6 weeks in all groups, then started to gradually decline over the 6-month period post-infection. Inpatients had the highest antibody titers, followed by HCWs, and then outpatients who had the lowest titers. Abbreviations: ELISA, enzyme-linked immunosorbent assay; HCW, health care worker; LME, linear mixed effect.

Antibody titers were measured at four time points: at baseline within 14 days of symptoms onset, 4-6 weeks, 3 months, and 6 months post-infection. Antibody titers reached the peak at 4-6 weeks, then started to gradually decline over the 6-month period across all three categories. Inpatients had the highest antibody titers of 6.23 ± 1.76 at 4-6 weeks that declined to 3.20 ± 1.53 at 6 months. The other groups had much lower antibody titers. HCWs had slightly higher antibody titers than outpatients at 4-6 weeks, 3.43 ± 1.79 and 3.18 ± 1.78, respectively, compared with 2.67 ± 1.94 and 1.71 ± 1.07 at 6 months for both groups, respectively (*P* <0.001) (Figure 1, Table 3).

**Table 3:** ELISA IgG titers by patients’ demography, comorbidities, and symptoms.

Variable	Baseline	4-6 weeks	3 months	6 months
	ELISA titer	ELISA titer	ELISA titer	ELISA titer
	Mean ± SD	Mean ± SD	Mean ± SD	Mean ± SD
**Group**				
Inpatient	3.28 ± 3.07	6.23 ± 1.76	6.07 ± 2.23	3.20 ± 1.53
Outpatient	1.47 ± 1.63	3.18 ± 1.78	2.57 ± 1.66	1.71 ± 1.07
Healthcare workers	_	3.43 ± 1.79	2.82 ± 1.38	2.67 ± 1.94
*P*-value	_	<0.001^a^	<0.001^a^	<0.001^a^
**Gender**				
Male	1.55 ± 1.63	4.86 ± 2.43	4.73 ± 2.76	3.21 ± 1.69
Female	1.81 ± 2.15	3.91 ± 2.02	3.50 ± 2.04	2.42 ± 1.56
*P*-value	0.464	<0.001^a^	<0.001^a^	0.004^a^
**Age group**				
13-40 years	1.22 ± 1.32	3.46 ± 2.06	2.94 ± 1.90	2.36 ± 1.59
41-59 years	2.36 ± 2.51	5.35 ± 2.06	5.22 ± 2.47	3.21 ± 1.70
≥60 years	2.85 ± 2.42	5.37 ± 1.95	5.79 ± 2.07	2.85 ± 1.35
*P*-value	0.002^a^	<0.001^a^	<0.001^a^	0.011^a^
**Common symptoms**				
**Fever**				
Yes	1.86 ± 2.37	4.90 ± 2.21	4.68 ± 2.44	2.90 ± 1.68
No	1.51 ± 1.22	3.14 ± 1.87	2.49 ± 1.67	2.25 ± 1.55
*P*-value	0.288	<0.001^a^	<0.001^a^	0.076
**Cough**				
Yes	1.86 ± 2.18	5.10 ± 2.12	5.03 ± 2.80	2.89 ± 1.54
No	1.58 ± 1.73	3.42 ± 2.06	2.80 ± 1.84	2.59 ± 1.88
*P*-value	0.444	<0.001^a^	<0.001^a^	0.150
**Comorbidities**				
**Obesity**				
Yes	2.10 ± 2.71	5.70 ± 2.09	5.41 ± 2.60	3.02 ± 1.50
No	1.64 ± 1.81	3.87 ± 2.12	3.47 ± 2.15	2.61 ± 1.76
*P*-value	0.536	<0.001^a^	<0.001^a^	0.076
**Hypertension**				
Yes	2.65 ± 2.68	5.49 ± 1.80	5.51 ± 2.43	3.31 ± 1.73
No	1.55 ± 1.77	4.04 ± 2.26	3.63 ± 2.31	2.58 ± 1.61
*P*-value	0.133	<0.001^a^	<0.001^a^	0.021^a^
**Diabetes mellitus**				
Yes	2.34 ± 2.59	5.52 ± 1.68	5.37 ± 2.19	3.12 ± 1.59
No	1.56 ± 1.75	4.03 ± 2.28	3.70 ± 2.40	2.65 ± 1.69
*P*-value	0.191	<0.001^a^	<0.001^a^	0.139
**Chronic lung diseases**				
Yes	1.66 ± 0.38	6.13 ± 2.33	6.50 ± 2.26	3.29 ± 1.22
No	1.70 ± 1.96	4.22 ± 2.21	3.91 ± 2.40	2.75 ± 1.69
*P*-value	0.969	<0.001^a^	<0.001^a^	0.379
Chronic kidney disease				
Yes	2.93 ± 2.85	4.86 ± 1.95	3.90 ± 2.29	2.85 ± 1.06
No	1.61±1.85	4.30 ± 2.26	4.03 ± 2.46	2.78 ± 1.69
*P*-value	0.064	0.418	0.879	0.92

a Statistically significant; test: one-way analysis of variance, independent samples Student’s *t*-test. Abbreviations: ELISA, enzyme-linked immunosorbent assay; IgG, immunoglobulin G.

Male patients had higher titers that were comparable at 4-6 weeks and 3 months (4.86 ± 2.43 and 4.73 ± 2.76, respectively) that declined at 6 months post-infection. Older patients, aged ≥41 years, had higher antibody titers than those aged 13-40 years. Although patients aged 41-59 years and 13-40 years peaked at 4-6 weeks, with antibody titers of 5.35 ± 2.06 and 3.46 ± 2.06, respectively, patients aged ≥60 years peaked at 3 months, with higher antibody titers of 5.79 ± 2.07 compared with the other groups (*P* <0.001) (Table 3).

Patients with fever and cough had significantly higher antibody titers that peaked at 4-6 weeks (4.90 ± 2.21 and 5.10 ± 2.12, respectively), followed by a marked decline at 6 months, than those who did not have these symptoms (*P* <0.001) (Table 3).

Patients with comorbid conditions, such as obesity, DM, HTN, asthma, and CKD, had higher antibody titers than those without these comorbidities. Patients with asthma had antibody titers that peaked at 3 months, with the highest antibody titers of 6.50 ± 2.26, compared with other comorbidities. Antibody titers in patients with HTN also peaked at 3 months (5.51 ± 2.43), whereas patients with obesity and patients with DM peaked at 4-6 weeks, with antibody titers of 5.70 ± 2.09 and 5.52 ± 1.68, respectively (*P* <0.001). Patients with CKD had lower antibody titers than those with previous comorbid conditions that peaked at 4-6 weeks, with titer levels of 4.86 ± 1.95 (*P* = 0.418). By 6 months post-infection, antibody titers in patients with these comorbidities significantly declined to levels comparable to those without comorbidities (Table 3).

### Course of illness and outcomes

Although 65% (80 of 123) of inpatients required oxygen therapy via simple face masks, non-rebreather masks, or non-invasive ventilation, only 20.3% (25 of 123) of them had evidence of pneumonia or ARDS on chest radiography. Of the 123 inpatients, 7.3% (9 of 123) required intubation and mechanical ventilation. Among these, 6.5% (eight of 123) were admitted to the intensive care unit (ICU) for severe COVID-19 pneumonia, and one patient died before ICU transfer.

Only 1.7% (2 of 120) of HCWs required admission for oxygen therapy, with evidence of pneumonia or ARDS on chest radiography, compared with none of the outpatients (*P* <0.001). Acute kidney injury and transaminitis were reported more frequently among inpatients (32.5% [40 of 123] and 35.8% [44 of 123], respectively). Death occurred in one (0.8%) of those admitted to the ICU, and one (0.9%) of the outpatients died 2 months after COVID-19 infection due to advanced pancreatic cancer (Table 4).

**Table 4:** Clinical course and outcomes.

Variable	Inpatients	Outpatients	Healthcare workers
	123	113	120
	n (%)	n (%)	n (%)
Respiratory support			
Simple oxygen/non-rebreather mask/non-invasive ventilation	80 (65.0%)	_	2 (1.7%)
Intubation	9 (7.3%)	_	0
Acute kidney injury	40 (32.5)	2 (1.8)	0 (0)
Transaminitis	44 (35.8)	6 (5.3)	1 (0.8)
Admission if Outpatient/health care worker		0 (0)	2 (1.7)
Intensive care unit admission	8 (6.5)	0	0
Death	1 (0.8)	1 (0.9)	0 (0)

Abbreviation: n, number.

## Discussion

To the best of our knowledge, this is the first study in Oman and the Gulf region to compare SARS-CoV-2 antibody responses across diverse population groups, including inpatients with severe disease, outpatients with mild symptoms, and HCWs. Although numerous studies have investigated antibody responses to SARS-CoV-2, most focused on specific populations, with small sample sizes and limited follow-up beyond 3 months post-infection [4]. Using an LME model to account for intra-individual variations, our study was able to explore factors influencing antibody dynamics more robustly.

We found that inpatients with severe disease were generally older, more likely to be male, and had higher rates of comorbidities, including obesity, DM, HTN, chronic lung diseases, and CKD than outpatients and HCWs. This is consistent with earlier studies reporting that older age, male gender, and comorbidities were associated with more severe disease [1,15,16]. However, some studies have noted inconsistent associations between male gender and disease severity [17].

Fever was most frequently reported symptom across all groups, followed by cough, dyspnea, myalgia, and fatigue, particularly, among inpatients. This aligns with previous studies reporting similar symptom profiles [16,17]. Although we did not assess symptoms duration, other studies have shown that prolonged symptoms, such as persistent cough, dyspnea, and fatigue, are associated with a higher risk of hospitalization [18,19].

Antibody persistence after infection with other coronaviruses (SARS-CoV and Middle East Respiratory Syndrome Coronavirus "MERS-CoV") has been documented to last up to 2 years [20]. However, reports on SARS-CoV-2 antibody durability have been inconsistent, with some showing rapid decline within 3 months and others reporting more sustained responses [9,10,21]. In our study, inpatients with more severe disease had higher antibody titers at all time points, followed by HCWs, with outpatients showing the lowest titers. Antibody levels peaked at 4-6 weeks post-infection in all groups, followed by a gradual decline over 6 months. We observed higher antibody titers in older patients, males, those with fever and cough, and those with comorbidities (asthma, DM, obesity, HTN, and CKD).

These findings are consistent with weak but growing evidence suggesting that older age, symptomatic disease, and greater severity correlate with stronger antibody responses [10,22,23]. However, some studies have reported limited correlations between antibody response and factors such as gender, comorbidities, and symptomatology [8]. Earlier research also showed that a subset of asymptomatic and mildly symptomatic patients remained seronegative for months post-infection compared with those with severe disease who consistently seroconverted [22,23]. Several previous studies, often with similar cohorts, reported that patients with severe disease—including those requiring ICU care—had delayed but higher and more sustained IgG responses than milder or asymptomatic cases, likely due to greater antigenic stimulation [4,10,11,15,24]. Our findings of more rapid antibody decline in outpatients are consistent with these observations. Similarly, Peghin *et al.* (2021) identified older age, greater symptom burden, and disease severity as predictors of longer-lasting immunity [7].

We also found that HCWs had higher antibody titers than outpatients but lower than inpatients, likely reflecting repeated occupational exposures to patients with high viral loads, as reported in seroprevalence studies from Spain and Oman [25,26]. Antibody durability was influenced by factors such as age, obesity, and HTN, whereas findings on DM were inconsistent, potentially reflecting differences in glycemic control and the impact of hyperglycemia on immune and inflammatory responses [23].

Finally, large-scale studies from the UK reported IgG-S antibodies, detectable in 92% of patients at 6 months, and IgG-N antibodies persisting in 72% at 18 months. However, limitations such as lack of PCR confirmation and severity stratification may have led to overestimation of seroconversion, especially in asymptomatic or mild cases [27].

## Conclusion

In this prospective cohort study, we found that advanced age; severe COVID-19 disease course; and comorbidities, particularly, obesity, DM, HTN, and asthma, were associated with higher and more persistent antibody responses over 6 months post-infection. Symptomatic patients, especially those with fever and cough, also mounted stronger responses. Antibody titers peaked at 4-6 weeks in all groups, with gradual decline thereafter.

### Strengths and limitations

To the best of our knowledge, this is the first study in the Gulf region that evaluated SARS-CoV-2 antibody responses across diverse groups of populations, including inpatients, outpatients, and HCWs. Our study included a relatively large sample size compared with previous studies with diverse demographic characteristics, which enhances the representation of the population in our region; therefore, it provides unique insights to the region’s epidemiologic data. Another strength of our study is the use of the LME model that accounted for intra-individual variability and time-dependent changes. This robust approach appropriately addressed missing data and the distributional characteristics of the data set, thereby enhancing the validity of our findings.

This study has several limitations. Neutralizing antibody and T-cell responses were not assessed; therefore, long-term protective immunity could not be determined. The follow-up period was limited to 6 months, providing insights on medium-term antibody persistence but not on long-term dynamics up to 18-24 months. Importantly, the study was conducted before widespread SARS-CoV-2 vaccination and before the emergence of later variants; therefore, reinfection and vaccination—two critical factors influencing antibody strength and durability—were not captured. Despite these limitations, the study provides essential baseline data on post–COVID-19 antibody responses in the Gulf region during the early pandemic period and highlights the influence of host factors and disease severity on antibody dynamics.

## Declaration of competing interest

The authors have no competing interests to declare.
